# Point-of-care Echocardiogram as the Key to Rapid Diagnosis of a Unique Presentation of Dyspnea: A Case Report

**DOI:** 10.5811/cpcem.2020.5.47012

**Published:** 2020-07-30

**Authors:** Michael Moore, Brian Dilcher, Joseph Minardi, Kimberly Quedado, Erica Shaver

**Affiliations:** West Virginia University School of Medicine, Department of Emergency Medicine, Morgantown, West Virginia

**Keywords:** Emergency Medicine, Point of Care Ultrasound, Dyspnea

## Abstract

**Introduction:**

Dyspnea is commonly evaluated in the emergency department (ED).The differential diagnosis is broad. Due to the large volume of dyspneic patients evaluated, emergency physicians (EP) will encounter uncommon diagnoses. Early, liberal application of point-of-care ultrasound (POCUS) may decrease diagnostic error and improve care for these patients.

**Case Report:**

We report a 48-year-old male presenting to the ED with cough and progressively worsening dyspnea for 11 months after multiple healthcare visits. Using POCUS, the EP was immediately able to diagnose a severe dilated cardiomyopathy (DCM) with left ventricular thrombus.

**Conclusion:**

Given that non-ischemic DCM is one of the most common etiologies of heart failure, often presenting with respiratory symptoms, POCUS is key to rapid diagnosis and, along with modalities such as electrocardiography and chest radiograph, should be standard practice in the workup of dyspnea, regardless of age or comorbidities.

## INTRODUCTION

In the emergency department (ED), healthcare providers are responsible for ruling out life-threatening causes of chief complaints. Of the more than 145 million ED visits in the United States in 2016, dyspnea accounted for 2.4%, or roughly 3.4 million visits.^1^ The differential diagnosis of dyspnea is broad, including both life-threatening and less urgent etiologies. Clinching the final diagnosis is guided by the clinical history, physical exam, and ancillary testing, including point-of-care ultrasound (POCUS). We discuss a case that highlights the importance of early POCUS use, specifically echocardiogram, in the ED for a patient with dyspnea.

## CASE REPORT

A 48-year-old male presented to the ED with complaint of cough and worsening dyspnea on exertion (DOE). Specifically, he was told he had an elevated D-dimer and troponin that were confirmed the day before during outpatient laboratory testing. The patient had been seen the previous day by the pulmonology clinic due to chronic cough and DOE for 11 months. At that time, he had blood work (including D-dimer and troponin) and a computed tomographic pulmonary angiogram (CTPA), which was negative for pulmonary embolism or gross cardiac abnormality, but showed bilateral ground-glass opacities consistent with pulmonary edema or pneumonitis. Specifically, the patient’s CTPA results indicated a normal heart size without pericardial effusion or evidence of right heart strain, without mention of cardiomegaly or visualized cardiac thrombus. Given the abnormal labs resulted after his discharge home from the pulmonology clinic, when his D-dimer and troponin were reported as abnormal, he was called by the pulmonologist who requested return to the ED for further evaluation.

On arrival to the ED, the patient stated he was experiencing worsening DOE and mild chest pressure. He denied any radiation, pleuritic, or positional components of the pressure. He also denied any lightheadedness, syncope, diaphoresis, nausea, pain or swelling in his lower extremities, orthopnea, or paroxysmal nocturnal dyspnea.

On chart review, we learned the patient had been seen in the lower acuity area of our ED six weeks prior for his chronic cough and reported DOE. His workup included a negative chest radiograph (CXR) and computed tomography of the chest with intravenous contrast. Given the patient’s lack of chest pain and previous “clean” health history, with complaint of cough, no further evaluation was completed during the initial ED presentation. With a negative workup at that time, the patient was advised to take an antihistamine for chronic sinusitis and referred to the pulmonology clinic for further evaluation. Of note, the patient was also seen by otolaryngology during the course of his symptoms and had a negative nasopharyngeal scope.

The patient had no other significant past medical or surgical history and no known drug allergies. His medications included albuterol, cetirizine, ipratropium, montelukast, omeprazole, tiotropium, prednisone, and over-the-counter testosterone supplements. Of note, these respiratory, gastric reflux, and seasonal allergy medications had only been added in recent months in attempts to treat his ongoing symptoms of cough and DOE. He denied any smoking history or recreational drug use; he did admit to consumption of three to five alcoholic beverages per day. He was employed as a commercial airline pilot and, prior to the onset of these symptoms, he was quite active and exercising daily. His family history was negative for venous thromboembolism, early cardiac disease, pulmonary disease, or aortic disease.

**Image 1:**
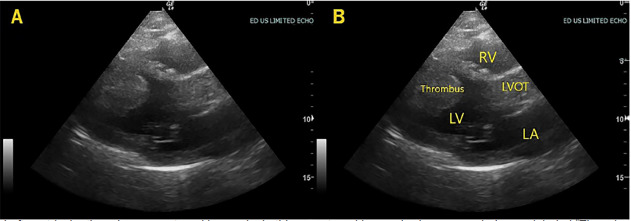
Left ventricular thrombus, parasternal long axis. In this parasternal long-axis view, a rounded mass labeled “Thrombus” in frame B is seen in the apex of the left ventricle. *RV*, right ventricle; *LVOT*, left ventricular outflow tract; *LA*, left atrium, *LV*, left ventricle.

**Image 2:**
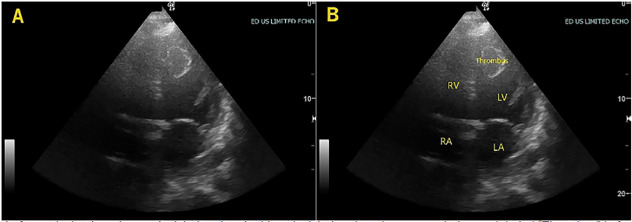
Left ventricular thrombus, apical 4-chamber. In this apical 4-chamber view, a rounded mass labeled “Thrombus” in frame B is seen in the apex of the left ventricle (LV). *RV*, right ventricle; *RA*, right atrium; *LA*, left atrium.

The patient’s vital signs were significant for an oxygen saturation of 88% on room air, heart rate 108 beats per minute, and blood pressure 138/90 millimeters of mercury. On physical exam, he was a healthy appearing, middle-aged male with conversational dyspnea. Head and neck exam were largely unremarkable. Chest exam revealed rales present in the mid and lower lung fields, bilaterally. Heart exam revealed a tachycardic rate with a regular rhythm and no murmurs or gallops. Lower extremities were symmetric, non-edematous, and non-tender bilaterally. Distal pulses were intact. Skin and neurological exams were normal. Given his hypoxia and conversational dyspnea, he was placed on supplemental oxygen and a point-of-care echocardiogram was performed by the emergency physicians (EP) (Image 1 and 2, Video). POCUS revealed a dilated, globally hypokinetic left ventricle with a significantly reduced ejection fraction (EF). There was also a large, mobile, left ventricular (LV) mass initially concerning for neoplasm or thrombus. No pericardial effusion was visualized. It was evident that the EF was significantly reduced, estimated to be about 30%. Of note, the attending EP was a general EP without fellowship training or focused practice in POCUS who was supervising general emergency medicine residents.

**CPC-EM Capsule**

What do we already know about this clinical entity?

Patients with cardiomyopathy and acute heart failure often present with dyspnea, a common chief complaint with the potential for morbidity and mortality.

What makes this presentation of disease reportable?

Point-of-care ultrasound (POCUS) quickly clinched the diagnosis of acute heart failure in a patient with progressive dyspnea.

What is the major learning point?

POCUS, even in the most novice physician hands, can assist in the quick identification of normal vs abnormal cardiac findings and guide further workup and treatment.

How might this improve emergency medicine practice?

POCUS is a low-risk diagnostic tool with a potentially high yield that can be used in the emergent evaluation of patients with dyspnea.

Given these findings, we discussed the case with the cardiology service. Cardiology suspected thrombus more likely than mass due to the acute, decompensated heart failure. Medical management was initiated, including a heparin infusion. The patient was admitted to the cardiology service. Upon admission, transthoracic echocardiogram (TTE) revealed an EF of 29% with a large, mobile LV mural thrombus. Coronary catheterization revealed minimal coronary artery disease. Cardiac magnetic resonance imaging showed an EF of 16%, LV mural thrombus, and evidence of LV non-compaction. The patient was ultimately diagnosed with non-ischemic dilated cardiomyopathy (DCM), of uncertain etiology. He was discharged home with a life vest, optimal medical therapy, and was advised to stop using alcohol as well as testosterone supplements. At three months post discharge he remained on medical therapy, including anticoagulation. He was found to have a persistently diminished EF of 30% without signs of LV thrombus on repeat TTE.

## DISCUSSION

This case serves to highlight the importance of the use of point-of-care echocardiography in the evaluation of dyspnea in the ED. This patient was evaluated multiple times over months by his primary care physician, emergency providers, otolaryngology, and pulmonology prior to his presentation to our ED for worsening cough and dyspnea. It was only then that he was accurately diagnosed with acute decompensated heart failure secondary to severe cardiomyopathy. Prior to his final diagnosis, his workup included CXRs, CTPA, nasopharyngeal laryngoscopy, and bronchoscopy. He had been treated for allergic rhinitis, asthma, gastroesophageal reflux disease, and chronic sinusitis with minimal to no improvement in his symptoms.

The diagnosis of heart failure in this patient, ultimately related to non-ischemic DCM, had likely not been strongly considered by previous providers. No echocardiogram had been performed and no documentation had suggested such a diagnosis on the differential. Providers were likely falsely reassured given the negative CTPA, his relatively young age, fit physical condition, and lack of other comorbidities. While he presented in acute decompensated heart failure on his second ED encounter, it is likely that he had a significantly reduced EF for some time. Had a point-of-care echocardiogram been performed earlier in his workup, it is likely there would have been a quicker final diagnosis. An earlier diagnosis may have led to earlier intervention and symptomatic improvement.^2^,^3^ There is some evidence that earlier recognition and initiation of therapy may slow progression of heart failure and reduce adverse events.

According to the American Heart Association, the incidence and prevalence of DCM has been challenging to predict based on multiple geographic and patient demographic variables, In most multicenter trials regarding heart failure, approximately 30–40% of patients have non-ischemic cardiomyopathy identified as the etiology.^4^,^5^ Evidence continues to support that sudden cardiac death (SCD) is a leading cause of mortality worldwide, and in up to 20% of these SCD cases, non-ischemic cardiomyopathies are to blame.^6^ Therefore, physicians on the front lines caring for the undifferentiated patient must recognize the warning signs of DCM when present. While cardiomyopathy is a less common cause of DOE in younger, healthier populations, it is nonetheless essential to consider in the differential diagnosis of DOE and cough. The potential for delayed diagnosis, significant morbidity, and even mortality is significant and very impactful in otherwise young, healthy patients. Although the etiology of this patient’s cardiomyopathy was not clearly identified, risk factors seem to be moderate alcohol use and testosterone supplementation, which has been noted to be a potential impetus for cardiomyopathy.^7^

POCUS was the key to a quick diagnosis in this protracted case of dyspnea. Thus, POCUS should be an essential part of the ED workup for a patient with dyspnea, regardless of patient age or comorbidities.^8^ Previous studies have demonstrated that POCUS performed by emergency medicine residents is comparable to echocardiography performed by cardiologists.^9^ Even in the hands of a non-POCUS focused EP, it was evident upon first glance that the heart in this case was abnormal. We feel that any EP with the most basic emergency echocardiography education would have identified the “abnormal” large mass/thrombus visualized in this case. For this particular patient, point-of-care echocardiogram assisted in rapid diagnosis of a previously overlooked etiology of dyspnea and the quick development of a treatment plan.

## CONCLUSION

This case emphasizes the vigilance EPs must maintain in all patients with cardiorespiratory symptoms. It is yet another illustration of the utility of POCUS to more thoroughly explore a broad, high-risk differential and provide a rapid, accurate diagnosis. Its early utilization in symptomatic patients should reduce diagnostic error and may lead to improved outcomes.

## Supplementary Information

Video Dilated cardiomyopathy (DCM) with left ventricular (LV) apical thrombus. In this brief, narrated video, the findings of a DCM are seen, including a dilated, globally hypokinetic LV, with poor mitral opening, as well as a rounded mobile mass in the LV apex consistent with thrombus.
